# Testicular Stiffness and Volume in Varicocele Patients: A Prospective Comparative Shear Wave Elastography Study

**DOI:** 10.3390/diagnostics15172150

**Published:** 2025-08-26

**Authors:** Ali Salbas, Raşit Eren Büyüktoka

**Affiliations:** 1 Department of Radiology, Izmir Katip Celebi University, Ataturk Training and Research Hospital, Izmir 35150, Turkey; 2 Department of Radiology, Foca State Hospital, Izmir 35680, Turkey; rasiterenbuyuktoka@hotmail.com

**Keywords:** varicocele, shear wave elastography, testicular stiffness, infertility, testicular volume, venous diameter, semen analysis, ultrasonography

## Abstract

**Background/Objectives**: A varicocele is a common, treatable cause of male infertility involving testicular alterations. Shear wave elastography (SWE) is a noninvasive imaging technique that may detect testicular changes. This study aimed to assess the testicular volume and stiffness (using SWE), compare them with healthy controls, and examine the associations with venous diameter and semen parameters. **Methods**: This prospective comparative study included 69 patients with a varicocele and 76 healthy controls. The testicular stiffness and volume were measured and compared among three groups: testes with a varicocele (Group A), contralateral healthy testes of the same patients (Group B), and testes of healthy individuals (Group C). In varicocele patients, the semen analysis results were compared with testicular stiffness and volume. The statistical analyses included an ANOVA, Kruskal–Wallis tests, a correlation analysis, and a receiver operating characteristic curve analysis. **Results**: Group A showed a significantly lower testicular volume compared to Group B (*p* = 0.004) and Group C (*p* < 0.001), and significantly higher SWE values compared to both groups (*p* < 0.001). No significant differences were observed between Group B and Group C (volume: *p* = 0.642; SWE: *p* = 0.094). In oligospermic varicocele patients, the testicular stiffness tended to be higher, though the difference was not significant (*p* = 0.051). The correlations between the testicular stiffness and volume (Group A: *p* = 0.488; Group B: *p* = 0.872; Group C: *p* = 0.222), between the venous diameter and stiffness (*p* = 0.067), and between the venous diameter and testicular volume (*p* = 0.245) were not statistically significant. The SWE cut-off value of 9.26 kPa provided a sensitivity of 73.1% (95% CI: 62.3–81.7%) and a specificity of 79.6% (95% CI: 72.5–85.2%) for identifying varicoceles. **Conclusions**: Varicoceles are associated with an increased testicular stiffness and a decreased volume, which may indicate possible structural parenchymal alterations. Shear wave elastography appeared to be capable of noninvasively detecting these changes and may have potential as a quantitative adjunct tool for evaluating testicular parenchymal changes in varicocele patients. However, further studies are required to validate these preliminary findings.

## 1. Introduction

A varicocele is a common pathology in men, characterized by the abnormal dilatation of the pampiniform plexus veins that form the spermatic cord [1]. It is considered one of the most common potentially correctable causes of male infertility [2]. Varicoceles are observed in approximately 15% of the general male population [1,2]. However, this rate increases to 40% in men with primary infertility and up to 75–80% in cases of secondary infertility [2,3]. The effects of varicoceles on the testicular parenchyma are explained by multifactorial mechanisms, including an increased testicular temperature, oxidative stress, the reflux of adrenal metabolites, and hormonal dysfunction [2,4,5].

Although the diagnosis of a varicocele is generally based on a physical examination, clinical evaluations largely depend on the clinician’s experience; therefore, ultrasonography (US) plays an important role in the diagnosis, especially in subclinical cases [2,6]. However, conventional US techniques may have limitations in detecting subtle or microscopic structural changes in the testicular parenchyma [7,8].

Shear wave elastography (SWE) is a novel, repeatable, and noninvasive ultrasonographic imaging technique used to assess the elasticity of various tissues. It has been studied for the evaluation of diseases in multiple organs, including the liver, breast, thyroid, and musculoskeletal system, and is gaining increasing attention in clinical practice [9,10,11,12,13]. Studies evaluating the testicular elasticity in patients with a varicocele are limited in number, and they have reported inconsistent findings: some have demonstrated an increased stiffness in the affected testes, while others have reported no significant differences compared to controls, and a few have even described decreased elasticity values [14,15,16]. These conflicting results may be attributed to limitations in patient populations, differences in measurement techniques, and methodological variations.

The aim of this study was to evaluate the testicular parenchymal elasticity using SWE in adult patients with a varicocele and to compare the obtained data with the data for both the contralateral testes and the testes of healthy individuals. In addition, possible relationships between the testicular volume, the elasticity values, and the diameter of dilated veins were investigated. Through this approach, the potential role of SWE as a noninvasive adjunct tool in evaluating possible varicocele-related parenchymal alterations was explored. Compared to the limited number of studies in the literature, this study represents, to our knowledge, the largest prospective investigation conducted in this field to date. In addition, our study integrated testicular stiffness, testicular volume, and semen parameters in the same prospective design, thereby providing a more comprehensive evaluation of the potential role of SWE in varicoceles.

## 2. Materials and Methods

Institutional ethics committee approval was obtained for the study (decision No: 0078, date: 13 February 2025). Written informed consent was obtained from all the participants prior to their inclusion in the study.

### 2.1. Study Design and Participants

**Figure 1:**
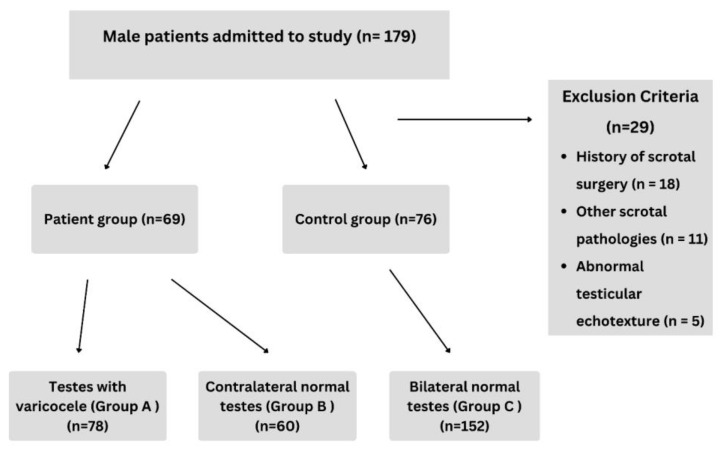
Flowchart of study enrollment population.

This prospective comparative study was conducted between March 2025 and July 2025. Male patients aged 18 years or older who were referred for scrotal ultrasonography and agreed to participate were eligible for inclusion. Patients with a history of scrotal surgery or with scrotal pathologies other than a varicocele (e.g., a testicular mass, a history of mumps orchitis or other testicular infections, a hydrocele, microlithiasis, or an abnormal testicular parenchymal echotexture on ultrasonography) were excluded from the analysis. The inclusion and exclusion criteria of the study are summarized in Figure 1. A total of 145 individuals (290 testes) were included in the study, consisting of 69 patients diagnosed with a varicocele (138 testes) and 76 healthy volunteer individuals (152 testes). A bilateral varicocele was present in 9 of the patients. A total of 78 testes with a varicocele were assigned to Group A, 60 healthy contralateral testes of these patients were assigned to Group B, and 152 healthy control testes were assigned to Group C [15,16]. The control group consisted of individuals who were referred for scrotal ultrasonography for non-varicocele-related indications (e.g., routine evaluation or non-specific scrotal complaints) and were randomly selected among those with no abnormalities detected on laboratory tests or scrotal ultrasonography. The same exclusion criteria were applied to both the patient and control groups.

In addition, the laboratory records of patients with a varicocele were reviewed. A semen analysis was not routinely performed in all patients, but was available for a subset of 43 patients. In these cases, the presence of oligospermia was evaluated. Oligospermia was defined as a sperm count of less than 15 million/mL [17].

### 2.2. Ultrasonography and Shear Wave Elastography Examination

All the ultrasonography and elastography evaluations were performed using a Samsung RS85 Prestige device (Samsung Medison Co., Ltd., Seoul, Republic of Korea) equipped with an LA2-14A high-frequency linear transducer. The examinations were conducted by a radiologist (A.S) with 8 years of experience in scrotal ultrasonography and 4 years of experience in SWE.

All of the cases were first evaluated using gray-scale ultrasonography, during which the testicular parenchymal structure, volume measurement, and exclusion criteria were assessed. The testicular volume was calculated by measuring the longitudinal, transverse, and anteroposterior dimensions in the supine position. For the volume calculation, the Lambert formula [volume = length × width × height × 0.71], as recommended by the Scrotal and Penile Imaging Working Group of the European Society of Urogenital Radiology (ESUR-SPIWG), was used [2]. The results are expressed in milliliters (mL).

**Figure 2:**
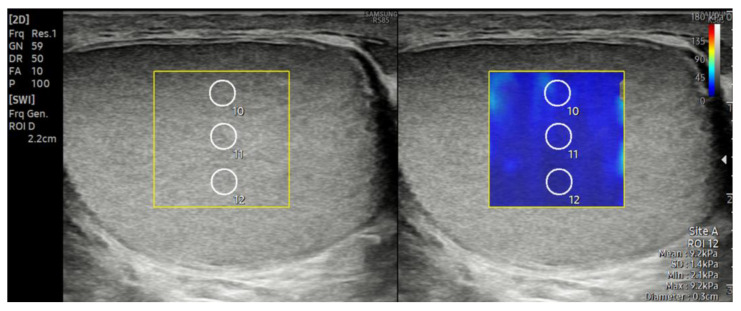
Shear wave elastography (SWE) image showing three regions of interest (ROIs, 3 mm in diameter) placed at the upper, middle, and lower poles of the testis in the longitudinal plane. As an example, ROIs from the middle pole are shown here. For each testis, a total of nine measurements (three per pole) were obtained to assess the parenchymal stiffness, and the mean value was calculated in kilopascals (kPa).

Subsequently, SWE examinations were performed on all the included cases. Measurements were obtained in the supine position while the patient was at rest, using sufficient gel and applying minimal probe pressure. For each testis, three measurements were taken from the upper, middle, and lower poles in the longitudinal plane, resulting in a total of nine measurements per testis (Figure 2). Similar to previous studies, a region of interest (ROI) with a diameter of 3 mm was used for the measurements [14,17]. The mediastinum testis was excluded from the ROIs. The average of the nine measurements obtained was recorded in kilopascals (kPa) to represent the testicular stiffness. To evaluate the reliability of the measurements, the reliability measurement index (RMI), which is automatically calculated by the device, was used. The RMI is an indicator that helps assess the accuracy of the elasticity measurement and ranges from 0.0 to 1.0. According to the device manufacturer, an RMI ≥ 0.4 is considered an acceptable threshold; however, in the literature, an RMI ≥ 0.8 is generally accepted as indicative of a reliable measurement [18,19,20,21]. In this study, only measurements with an RMI ≥ 0.8 were included in the analysis.

Finally, a varicocele evaluation was performed in the standing position using color Doppler ultrasonography. During the Valsalva maneuver, the diameter of the pampiniform plexus vein was measured at its widest segment, and the duration of reflux was assessed using both color and spectral Doppler US. In accordance with the ESUR-SPIWG recommendations, a vein diameter greater than 3 mm and the presence of reflux lasting more than 2 s during the Valsalva maneuver were considered diagnostic criteria [2].

### 2.3. Statistical Analysis

The normality of distribution for continuous variables was assessed using the Shapiro–Wilk test, and the homogeneity of variances was evaluated using Levene’s test. For data showing a normal distribution and homogeneous variances, a one-way analysis of variance (ANOVA) was used for comparisons between groups, and the Tukey HSD post hoc test was applied in cases where a significant difference was detected. For variables that did not show a normal distribution, the Kruskal–Wallis test was used, and pairwise group comparisons were performed using the Bonferroni-corrected Mann–Whitney U test. In varicocele cases with available semen analysis results (*n* = 43), the testicular volume was compared between individuals with oligospermia and those with normal sperm parameters using Student’s *t*-test, while the SWE values were analyzed using the Mann–Whitney U test. Patients without semen analysis results were excluded from these comparisons, and semen-related analyses were therefore limited to this subgroup. The relationships between continuous variables were evaluated using a Spearman correlation analysis. In addition, a multivariable linear regression analysis was performed in Group A (testes with a varicocele, *n* = 78) to assess potential confounders, with SWE as the dependent variable and age, vein diameter, and testicular volume as independent predictors. Additionally, a receiver operating characteristic (ROC) curve was generated to compare Group A and Group C, and the area under the curve (AUC), optimal cut-off value, sensitivity, and specificity were calculated. All the statistical analyses were performed using IBM SPSS Statistics for Windows, version 28.0 (IBM Corp., Armonk, NY, USA). A *p*-value of <0.05 was considered the threshold for statistical significance.

## 3. Results

A total of 145 male individuals were included in the study, of whom 69 had a varicocele and 76 were healthy controls. Among those with a varicocele, 60 individuals (87%) had a left-sided varicocele only, while 9 (13%) had a bilateral varicocele. No cases of an isolated right-sided varicocele were observed. The total number of testes evaluated was 290, consisting of 78 testes with a varicocele (Group A), 60 healthy contralateral testes from the same patients (Group B), and 152 testes from healthy individuals (Group C).

An evaluation of the semen analysis records of the 69 patients with a varicocele revealed that 16 patients (23.2%) had oligospermia, 27 (39.1%) had normal sperm parameters, and 26 (37.7%) had no available semen analysis data. Among the 16 patients with oligospermia, 12 had a left-sided and 4 had a bilateral varicocele.

In Group A (testes with a varicocele), the mean diameter of the pampiniform plexus vein was 3.53 ± 0.59 mm, with measurements ranging from 3.00 to 5.60 mm.

The mean age was 36.27 ± 14.51 years in Group A, 38.52 ± 15.60 years in Group B, and 39.09 ± 14.00 years in Group C (Table 1). There was no statistically significant difference in age among the three groups (*p* = 0.146).

The mean testicular volume was measured as 21.07 ± 3.69 mL in Group A, 23.37 ± 4.62 mL in Group B, and 23.94 ± 4.17 mL in Group C (Table 1). The testicular volume in Group A was significantly lower compared to both Group B (*p* = 0.004) and Group C (*p* < 0.001). No significant difference in testicular volume was observed between Group B and Group C (*p* = 0.642).

The mean SWE value was calculated as 10.42 ± 2.50 kPa in Group A, 7.73 ± 2.13 kPa in Group B, and 8.09 ± 1.59 kPa in Group C. Group A had significantly higher SWE values compared to both Group B (*p* < 0.001) and Group C (*p* < 0.001). No significant differences in the SWE values were observed between Group B and Group C (*p* = 0.094).

**Figure 3:**
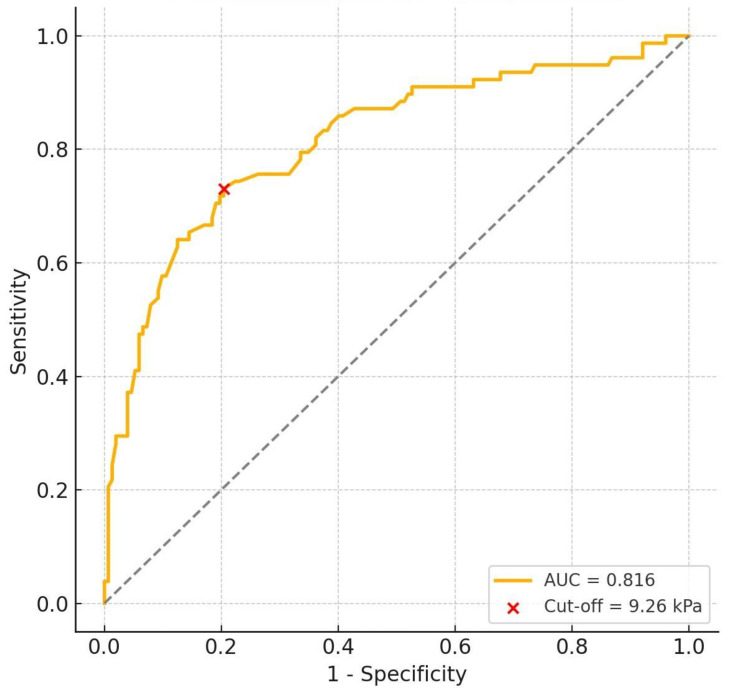
Receiver operating characteristic (ROC) curve showing the diagnostic accuracy of SWE in differentiating Group A (testes with a varicocele) from Group C (healthy testes). The calculated AUC was 0.816. A cut-off value of 9.26 kPa yielded a sensitivity of 73.1% and a specificity of 79.6%.

In the ROC analysis based on SWE values between Group A (testes with varicocele) and Group C (healthy testes), the area under the curve (AUC) was 0.816 (95% CI: 0.753–0.875). The optimal cut-off value for the diagnosis of a varicocele was determined to be 9.26 kPa, yielding a sensitivity of 73.1% (95% CI: 62.3–81.7%) and a specificity of 79.6% (95% CI: 72.5–85.2%) at this threshold (Figure 3). This threshold reflects the discriminatory capacity of SWE between testes with a varicocele and healthy testes.

In the evaluation of only the testes with a varicocele in the 43 patients with available semen analysis results, no statistically significant differences were found in the testicular volume (*p* = 0.184) or SWE value (*p* = 0.051) between individuals with oligospermia (*n* = 16) and those with normal sperm parameters (*n* = 27) (Table 2).

No significant correlation was found between the testicular stiffness and volume in any of the groups (Group A: r = −0.080, *p* = 0.488; Group B: r = −0.021, *p* = 0.872; Group C: r = −0.100, *p* = 0.222). Although a positive trend was observed between the vein diameter and SWE in Group A, the correlation was not statistically significant (r = 0.208, *p* = 0.067). Similarly, no significant correlation was found between the vein diameter and the testicular volume (r = −0.133, *p* = 0.245).

To account for potential confounding factors, a multivariable linear regression analysis was performed in Group A (testes with a varicocele, *n* = 78), with age, vein diameter, and testicular volume included as predictors of SWE values. None of the following variables were independently associated with SWE: age (β = −0.009, *p* = 0.646), vein diameter (β = 0.318, *p* = 0.521), and testicular volume (β = −0.073, *p* = 0.357). The overall model did not significantly explain the variability in the SWE values (adjusted R^2^ = −0.02, *p* = 0.680).

## 4. Discussion

In this prospective comparative study, the testicular volume and parenchymal elasticity were evaluated using SWE in adult patients with a varicocele. The findings indicate that SWE may provide complementary information in the evaluation of parenchymal characteristics in testes with a varicocele. In our study, testes with a varicocele showed a significant reduction in volume along with significantly increased SWE values compared to both contralateral non-varicocele testes and non-varicocele control group testes.

In the literature, varying results have been reported regarding the effect of varicoceles on the testicular volume. Most adult series have indicated that the volume of the affected ipsilateral testis is significantly lower compared to that of healthy testes [15,16,22]. In contrast, some studies have reported no significant differences in either the ipsilateral or contralateral testicular volumes compared to the control group [14,23]. In a study involving 2080 young adults, a significant volume loss was reported on the varicocele side, while possible compensatory hypertrophy was observed in the contralateral testis [24]. On the other hand, some studies focusing on the adolescent population have suggested that a high-grade varicocele may lead to a bilateral volume reduction over time [25]. This variability may be attributed to differences in patient age range, varicocele grade, and sample size.

Our study demonstrates that, in the adult population, a varicocele primarily leads to a reduction in volume in the ipsilateral testis. The mean volume of varicocele-affected testes was significantly lower than that of both the contralateral testes in the same patients and the testes of healthy controls. In contrast, no significant difference was found between the volumes of contralateral testes and those of healthy individuals. These findings are consistent with the majority of data in the literature and support the notion that the impact of a varicocele on the testicular volume predominantly occurs on the ipsilateral side. The absolute testicular volume values in our study were higher compared to those reported in many previous series. In most earlier studies, the testicular volume was calculated using the ellipsoid formula (length × width × height × 0.52). However, evidence in the literature suggests that the Lambert formula (length × width × height × 0.71) provides results that more closely reflect the actual testicular volume [26,27]. Therefore, in accordance with the recommendations of the ESUR-SPIWG, the Lambert formula was used in our study [2]. This methodological difference may be the main reason for the discrepancy in the absolute volume values.

Studies investigating the relationship between varicoceles and testicular parenchymal stiffness have reported conflicting results. Many have shown that testes with a varicocele exhibit higher SWE values compared to healthy testes, suggesting that this may be attributed to fibrosis caused by chronic hypoxia, an increased temperature, and microvascular damage [16,17,22,28,29]. Additionally, some of these studies have reported an association between oligospermia and higher SWE values [17,29]. However, no significant correlation has been found between the testicular volume and elasticity [16]. On the other hand, certain studies have reported decreased elasticity values or no difference at all in testes with a varicocele [14,15,30,31]. It has been suggested in these studies that a decrease in testicular stiffness may be observed in the later stages of a varicocele due to germ cell apoptosis.

It is noteworthy that, in most of the cases reported in the literature with increased testicular stiffness, a reduction in the testicular volume was also present. Jedrzejewski et al. observed a significant increase in the SWE values only in testes with a varicocele that had experienced more than 20% volume loss, while no significant difference was found between cases with less volume loss and healthy individuals [32]. In this context, it is suggested that changes in the testicular parenchymal elasticity may reflect different stages of testicular damage.

In this study, the SWE values of testes with a varicocele were found to be significantly higher compared to both contralateral testes and the testes of healthy individuals. In contrast, no significant correlation was observed between the testicular volume and SWE. Additionally, a positive trend was observed between the vein diameter and the testicular stiffness in testes with a varicocele; however, this relationship was not found to be statistically significant. These findings indicate that the increase in elasticity may not be directly related to the testicular volume and could be associated with early parenchymal changes. Moreover, our study showed that the cut-off value of 9.26 kPa obtained through the ROC analysis could distinguish testes with a varicocele from healthy testes with a sensitivity of 73.1% and a specificity of 79.6%. This cut-off value should be interpreted as reflecting the discriminatory performance of SWE in this study cohort rather than a clinically validated diagnostic threshold. This cut-off value has not been externally validated, and its clinical relevance remains to be established. To the best of our knowledge, there is no clearly defined or widely accepted cut-off value established by SWE for the diagnosis of varicoceles in the English-language literature. From this perspective, the cut-off value of 9.26 kPa identified in our study contributes to the limited data currently available in this field. However, given the methodological limitations, including the sample size and the single-center design, our findings should be regarded as preliminary and hypothesis-generating. The validation of these results in larger, multicenter studies would be necessary before considering potential clinical applications.

The SWE values reported for the testis in the literature generally range between 2 and 10 kPa, and the mean kPa values observed in both the varicocele and control groups in our study were higher compared to those of many previous studies [14,15,17]. The SWE values in our study were similar only to those reported by Erdogan et al. [16]. This difference from some of the other studies may be related to methodological factors such as the device used, the transducer type, the measurement technique, and the elastography settings. In our study, the inclusion of only measurements with an RMI ≥ 0.8 enhanced the reliability of the elastography data. This approach is important for maintaining methodological consistency in SWE assessments and improving the interpretability of the findings. Large-scale, multicenter studies using different devices are needed to more clearly determine the impact of these methodological differences.

Significant associations between testicular elasticity and sperm parameters have been reported in the literature. In patients with a varicocele, the testicular stiffness has been shown to be higher in oligospermic individuals compared to normospermic individuals [17,29]. Similarly, studies conducted in infertile men have emphasized that an increased testicular elasticity may be associated with impaired sperm parameters [33,34]. Moreover, although the testicular volume was similar among the groups in these studies, the finding that an increased elasticity was associated with oligospermia suggests that this relationship may be independent of volume.

In this study, no significant difference in testicular volume was observed between the groups when evaluating varicocele patients with available semen analysis results. Although the testicular stiffness was higher in oligospermic individuals, this difference did not reach statistical significance (*p* = 0.051). Varicoceles are known to be associated with oligospermia; however, our study did not reveal significant differences in the testicular volume or SWE values between oligospermic and normospermic varicocele patients. This finding may suggest that the testicular volume and stiffness are not markedly different between these subgroups; however, two important considerations must be noted. First, the number of patients with an available semen analysis was relatively small, which may have limited the statistical power and prevented the observed trend in the SWE values from reaching statistical significance. Second, since all the individuals in this comparison had a varicocele, the absence of significant differences may have been due to the presence of underlying parenchymal alterations in both groups.

This study has several limitations that should be acknowledged. The first and most important limitation is that all ultrasonographic and elastographic evaluations were performed by a single radiologist. Although this eliminated interobserver variability, it also limits the generalizability of the findings, as the results may not be fully applicable to different operators or clinical settings. In addition, the intraobserver reproducibility was not formally tested through repeated measurements or statistical reliability analyses, which introduces potential bias and reduces the strength of the findings. Another limitation is the absence of histopathological confirmation. However, such assessments are generally impractical due to the procedural characteristics of varicocele surgery. Moreover, the clinical examination findings and the clinical grading of the varicoceles were not included, and the diagnosis was based solely on ultrasonographic criteria, which may have led to the inclusion of subclinical cases. In addition, although minimal pressure was applied with the probe during the SWE examinations, the effect of probe compression on the elasticity values cannot be fully excluded and represents a potential limitation of the technique. Furthermore, the subgroup analysis comparing oligospermic and normospermic patients was based on a limited sample size, which reduces the statistical power, and the results should be interpreted with caution. Finally, although the study was prospectively designed, it was conducted at a single center, and this further restricts the generalizability of the results. The findings should therefore be interpreted as preliminary and require validation in larger, multicenter cohorts with multiple operators to better establish their reproducibility and external applicability. Despite these limitations, this study represents the largest prospective study to date, based on sample size, to assess the testicular stiffness and volume in patients with a varicocele using shear wave elastography. In this context, the findings indicate that SWE may provide supportive information in the noninvasive evaluation of varicocele-related testicular changes. Nonetheless, its potential diagnostic role, particularly in subclinical cases, remains uncertain and should be clarified through larger, multicenter studies supported by histopathological validation.

## 5. Conclusions

In summary, this prospective, comparative study provides preliminary evidence that both testicular volume loss and an increased parenchymal stiffness may be detectable ultrasonographic features in adult patients with a varicocele. These observations, obtained using SWE, suggest that the technique may have a role in the noninvasive assessment of varicocele-related parenchymal alterations. The cut-off value of 9.26 kPa derived in this study population should be regarded as exploratory, as it has not been externally validated and its clinical significance remains to be determined. Overall, the findings should be considered hypothesis-generating, and their clinical applicability requires confirmation through larger, multicenter studies incorporating histopathological correlations and long-term reproductive outcomes.

## Figures and Tables

**Table 1:** Mean age, testicular volume, and SWE values of the testes according to the groups.

Group	Age (Year)	Volume (mL)	SWE Value (kPa)
A	36.27 ± 14.51	21.07 ± 3.69	10.42 ± 2.50
B	38.52 ± 15.60	23.37 ± 4.62	7.73 ± 2.13
C	39.09 ± 14.00	23.94 ± 4.17	8.09 ± 1.59
A + B + C	38.21 ± 14.48	23.05 ± 4.31	8.64 ± 2.26

Results are shown as mean ± SD. kPa: kilopascals, mL: milliliters, SD: standard deviation, SWE: shear wave elastography.

**Table 2:** Comparison of mean testicular volume and SWE values in varicocele patients based on semen parameters.

Semen Analysis	Testicular Volume (mL)	SWE (kPa)
Oligospermic (*n* = 16)	19.45 ± 2.89	10.99 ± 1.45
Normospermic (*n* = 27)	21.02 ± 4.08	10.10 ± 2.18
*p*	0.184	0.051

Note: Only varicocele patients with available semen analysis results were included. In patients with a left-sided varicocele, measurements from the affected (left) testis were used; in bilateral cases, the mean of both testes was used to represent a single value per patient. The results are presented as the mean ± standard deviation. kPa: kilopascals, mL: milliliters, SD: standard deviation, SWE: shear wave elastography.

## Data Availability

The data presented in this study are available from the corresponding author upon request. The data are not publicly available due to privacy and ethical restrictions.
